# Madness, as Treated by Shakspere

**Published:** 1849-10-01

**Authors:** R. H. Horne


### MADNESS, AS TREATED BY SHAKSPERE.

### A Psychological Essay.

BY R. H. HORNE.

Of all the diseases of the body and the mind, the one least understood is that condition which we call "madness." Certainly there is no other which presents half such wonderful and complicated phenomena. Madness is profoundly melancholy, heart-breaking, hopeless; and it is ferocious, terrific, exalted with monstrous hopes: madness may be most gentle and consistent, or it may be most violent and variable, alternating between appalling curses and the tenderest tears; it is often depressed beneath despair—yet far more commonly uplifted, and it generally ascends the highest heaven (or descends to the lowest hell) of "invention," according to the quality of its powers. Madness is most extravagant and irrational; yet is it reasoning from morning till night, even till it becomes the fool, not of REASON, but of the process of *reasoning*. In one sense, it is generally quite right; and you will see this, too, if you will only consent to look at the given question from the same point of vision. It is seldom able to deceive anybody who does not consent to it in the first instance, or only to deceive for a brief period; but it constantly deceives itself, which, however, makes no difference in the fact, the feeling, or the progress of the hallucination. Madness is tragic and pathetic, and this we thoroughly feel; it is also comic and fantastic to the last degree, but these things we do *not* feel, or at least we can take no part in the pleasantry. We can shed tears with it in true sympathy, but we cannot laugh with it cordially. A madman's jest makes you become grave and thoughtful. Madness is isolated, solitary, incapable of combination to aid or to be aided in a given course, its continuity being dependent on the winds, or other ungovernable elements of its own; it drifts alone upon its own surging waves—a wreck on its own mental sea. It seems to understand itself, though other minds do not understand it. Except in certain effects, physical condition, general habits, and outward peculiarities, even the most intellectual and experienced of those who have charge of lunatics, confess that they know little or nothing of this wonderful disease of the mind. Moreover, its ramifications and shades are almost universal. When Dr. Haslam, the celebrated author and physician, was cross-questioned as a witness in a court of law, he was asked how he distinguished an insane patient from a person of perfectly sound mind? He replied, "I consider that there is no *perfectly* sound mind, except the mind of the Deity."

The above opinion may have been carried to an extreme; nevertheless, it is not to be denied that, in the infinitude of shades and gradations of madness, a far greater number of individuals are more deeply "touched" than is at all suspected. Nor does this greatly signify in the current affairs and bustle of the world, where a marvellous balance is kept up amidst the ever-warring wheels, so that the half deranged wits who fall a prey to rogues, continually rise again on the ruins of those who have outwitted themselves by equally wild fallacies. Seeing all these things about us,—that degrees of madness are far more common than we deem, but that the highest degrees are rare, and still more wonderful to witness in those of extraordinary mental powers, or who have possessed striking characters before the edifice fell to ruin and desolation,—it will be deeply interesting to consider and study what Shakspere has done for us on this great and mysterious affliction of humanity. He has left his Book of Nature marked for us at several profoundly important places.

Nothing can be more subtly, yet decisively marked, than the gradations by which King Lear, from a venerable and almost doting old man,—who wishes to "shake all cares and business" from his bowed-down tree of life, so that he may "unburdened crawl towards death,"—rises with preternatural strength into the most towering condition of utter madness. We had a general impression, in recollections of the tragedy, that the first breaking forth of the madness of Lear—that is, the turning point when his rage and conflicting passions carried his mind beyond all self-government, or definite conception of itself and its own purposes—was displayed at the close of Act II., where he exclaims to his daughters,—

> No, you unnatural hags,
> I will have such revenges on you both,
> That all the world shall——*I will do such things*—
> *What they are, yet I know not;* but they shall be
> The terrors of the earth. You think I'll weep;
> No, I'll not weep:—
> I have full cause of weeping; but this heart
> Shall break into a hundred thousand flaws,
> Or ere I'll weep:—O Fool, I shall go mad!

Thus, after rising apparently to the highest pitch of fury with one unnatural daughter, he suddenly discovers another daughter yet more unnatural, so that his violence is impelled to burst all bounds: he then pauses an instant to contemplate the first daughter again, because he now believes her less monstrous, and by comparison almost kind, when he immediately perceives that she is even worse than the second daughter. These sudden and violent bursts and recoils of passion—the rapid alternations of the frenzy of hate, with loving though much-exacting hope — and the manifest confusion of his brain, as displayed in the equal fury and vagueness of his purposes, added to the final declaration of the frantic condition into which he was rushing—all these seemed to indicate the point where Lear's reason gave way, and every fresh emotion and thought *would* impel him deeper into the chaotic elements of insanity.

Coleridge places the first symptoms of "positive derangement" at the close of the 4th Scene of Act III., where the forlorn King tears off his clothes amidst the storm on the heath, and discourses with Edgar, who is disguised as Mad Tom. (See "Literary Remains," Vol. II.) We have given this opinion that consideration which such an authority as Coleridge must always claim; referring, however, to the tragedy for a close examination throughout, we cannot by any means coincide with him, even though the faithful Kent seems to entertain the same opinion, in this scene:—

> *Kent.* Importune him once more to go, my lord:
> His wits *begin* to unsettle.
>
> Act III., Scene 4.

Perhaps this remark of Kent's might have influenced, if not partly originated, the opinion of Coleridge. We are too apt to regard the remarks of the *dramatis personæ* in a play, concerning each other, as conclusive, and to form our judgments accordingly; whereas, each character is influenced by his own personal feelings and interests, passions and peculiarities; and however important an authority or witness on a given question, the opinion must only be taken in connexion with the rest we know of the characters, words, and *actions* of the individual under consideration. The entire work must be carefully consulted. Taking, then, Shakspere's tragedy in its entirety, we are led to turn back, not merely from the third Act, but from the second Act, and again from that to the first, until we are finally compelled to think that the madness of Lear is at least "prepared for" in the very first scene—if not something more.

It hence appears, that so subtly has Shakspere woven his web, that from the first scene to the last, he has "enmeshed them all," and we are consequently obliged to commence our study of his treatment of madness in the character of Lear, with the very first scene of the tragedy.

The character of Lear at the opening of the tragedy is that of a very old and venerable king, equally affectionate and choleric; magnificently generous, yet minutely selfish; ready to give away all substantial things, but exacting a measureless return of ideal good—all his land, and gold, and power, provided he have in exchange the utmost love, or at least the assertion of it, (for it is manifest that the inward fact was not of so much importance to him as the external form,) and that he also retain the name and dignity of a king. He has a largeness of heart and store of tenderness for those he loves, and he is readily pacified by a slight concession, even in his anger; but he is no doubt most overbearing and despotic in his feelings and notions of what is due to himself as a king and a father, and he would absolutely rule over the soul (as his subject, no less than the body) of his child. His exactions in this respect, and his general overweening sense of the kingly office, alternate between dotage and monomaniasm, and often, perhaps, are compounded of both.

In this state of mind, and when upwards of eighty years of age, Lear determines on laying down the cares of sovereignty, and accordingly makes the division of his kingdom, and summons his three daughters to be present at the ceremony of the announcement. Let it be noted, therefore, that the division has been already settled in the old King's mind, assuming the great love borne him by his daughters; so that his asking the question of how much they loved him, and which of them loved him most, in order that he might know to whom the largest portion ought to fall, was a mere caprice of selfish exaction, though not unkindly or invidiously intended. "The trial," says Coleridge, "is but a trick, and the grossness of the old King's rage is in part the natural result of a silly trick, suddenly and most unexpectedly baffled and disappointed." It was, perhaps, not very seriously intended; he made sure of all the answers, as matter of course; but directly he finds himself thwarted, then it begins to be a dreadfully serious matter with him; the more so that this comes from his favourite daughter, from whom he expected most. Nevertheless, he tries to get Cordelia to speak in the same strain as her sisters; it was only the declaration that he sought to enforce, and not any practical proof of her love. Cordelia, however, as Hazlitt acutely observes, has a dash of "her father's obstinacy in her," and refuses to comply with his will, even though she sees the old King's passions begin to boil up, and that he is becoming more furious every instant. There is a reprehensible and cruel hardness in the firm refusal of Cordelia to humour her aged father; still his rage is beyond all measure of rationality, when we consider the cause.

> *Lear.* Here I disclaim all my paternal care,
> Propinquity, and property of blood,
> And as a stranger to my heart and me,
> Hold thee, from this, for ever. The barbarous Scythian,
> Or he that makes his generation messes
> To gorge his appetite, shall to my bosom
> Be as well neighbour'd, pitied, and relieved,
> As thou my sometime daughter.
> *Kent.* Good my liege,—
> *Lear.* Peace, Kent!
> Come not between the dragon and his wrath!

There is something in the excess of this rage, under the given circumstances, which is beyond a passion, and can hardly be called sane. The very reason he gives for it, is a mad reason. He has "loved her most," and thought "to set his rest on her kind nursery,"—that is, to dwell with her. Yet because she has publicly thwarted his whim—his idle little plot—down come all these monstrous denunciations and disinheritance. Kent again interposes—the King threatens him with destruction; to which Kent replies—

> Be Kent unmannerly
> When Lear is mad.

This expression might not have been meant literally; but it is too strong, and too well suited to the occasion to be treated lightly. In Scene 4 of Act III., it will be recollected that Kent says of the King—

> His wits begin to unsettle.

But here in Scene 1, Act I., when Kent's own feelings are highly excited, and his character put to the test, he declares that Lear *is* mad. This is a good example of the need of previous caution against forming a judgment of a character in a drama entirely by what one or more of the other characters may say. In the present case, therefore, we are not to look to honest Kent for a decision concerning the commencement of Lear's madness, but to Shakspere.

"It may be here worthy of notice," says Coleridge, "that Lear is the only serious performance of Shakspere, the interest and situations of which are derived from the assumption of a gross improbability." This is an extraordinary assertion, and naturally calls up the oft-considered question of the gentle lady who fell in love with the black and war-scarred Moor; the supernatural influences in "Macbeth" and "Hamlet;" to say nothing of the gross quibble on the birth of Macduff, or of the pound of flesh without blood, in the "Merchant of Venice." Improbability is not the question, but the possible and natural; and in any case, the truth and nature developed, the premises being taken for granted. In "Lear," however, this very improbability seems to our minds rather an exception, and more a merit than a defect, since it serves to show the condition of the old King's mind, its vigorous eccentricities, and extravagant exactions. Such a condition, if not already there, is easily brought to the borders of madness. This is finely displayed in the first scene of the tragedy. Kent persists in his remonstrance, notwithstanding that the old King has threatened his life three several times, if not four. ("Come not between the dragon and his wrath!" —"The bow is bent and drawn, make from the shaft!"—"Kent, on thy life, no more!"—"O, vassal miscreant!") The King finally declaring, when he banishes Kent, that neither his *nature* nor his *place* can bear it. He is thus shown to be brimfull of the regal office, even though he has resigned it, and to be conscious of the ungovernable character of his own passions. His youngest daughter, hitherto most beloved, is now "newly adopted to his hate;" "dowered with his curse;" and "made a stranger by his oath;" and thus ends his first Scene in the first Act, wherein, to our thinking, the key-note of *coming* madness is struck in the most prelusive and masterly manner.

In Scene 3 of this first Act, we hear that Lear has "struck a gentleman for chiding of his Fool;" this person being a gentleman attendant on Goneril. In Scene 4, a knight remarks to the King that since Cordelia's departure for France, "the Fool hath much pined away." The affectionate nature of the Fool, and the reproach unintentionally implied by the contrast of this affection with his own obduracy towards Cordelia, evidently touches him painfully—

*Lear.* No more of that: I have noted it well.

He had observed it with twinges of conscience, and cannot bear to be reminded of it. His dissatisfaction with himself thus adds weight to his previous state of dissatisfaction at the want of respect to himself and his followers, and he immediately vents it on the steward, whom he strikes.

The breaking up of the sense of personal identity, and the confusion of self-consciousness in madness, is finely shadowed forth in the following speech—

*Lear.* Does any here know me? Why, this is not Lear. Does Lear walk thus?—speak thus? Where are his eyes? Either his notion weakens, or his discernings are lethargied. Sleeping or waking? Ha! sure 'tis not so.—Who is it that can tell me who I am?

This is not all said in pretended astonishment and irony. His mind is shaken, and he feels it. To his last question, the Fool answers—"Lear's shadow." The King does not hear him, but continues his inquiry as to who he is.

*Lear.* I would learn that: for by the marks of *sovereignty*, knowledge, and *reason*, I should be *false* persuaded I had daughters.

Goneril perceives something very strange about her father, but her utter hardness of heart causes her to make light of it.

*Lear.* Your name, fair gentlewoman?
*Gon.* Come, sir;
This admiration is much o' the favour
Of other your new pranks.

She uses the term "admiration" in the sense of bewilderment, and alludes to sundry new pranks committed in a similar state of mind, in some of which it seems quite likely the old King had really indulged. The violent passion into which Lear is thrown by this speech of Goneril, causes him to say in an agony of self-reproach—

O most small fault,
How ugly didst thou in Cordelia show,
Which like an engine, *wrench'd my frame of nature*
*From the fix'd place.*

It is very plain from all these things, that the expression of "his wits begin to unsettle," must, so far as the fact is concerned, be applied to him at a much earlier period than where it is used; indeed, we should apply it to this very point of his sudden hatred and curse of Cordelia—not, be it observed, because of what he now says of this himself, but because it was the first sign of an aberration of reason, resolute in act as if it had been founded on substantial premises. He afterwards declares that when Regan shall hear of Goneril's wickedness—" with her nails she'll flay thy wolfish visage." Another sign of madness is apparent in all these recourses and references to personal violence, and in an old man of eighty. Already in this first Act, Lear has four times threatened a man's life, struck a gentleman of the court, struck Goneril's steward, and now threatens one daughter with the personal fury of another in his cause.

In Scene 5, being the last of Act I., the old king laughs wildly; his thoughts are wandering about Cordelia, (where he says abstractedly from all that is going on—" I did her wrong;") and at the close he bursts out with an anguish of burning consciousness in his brain—

O, let me not be mad—not mad, sweet heaven!
Keep me in temper—I would not be mad!

The tragic pathos of this, together with the hasty demand, if the horses are ready, which properly concludes the first Act, is as profoundly affecting as the psychological development of the progress of insanity is worthy of the most serious study. When Coleridge notices " the mind's own anticipation of madness," he seems to us not to go far enough, since, in our view, Lear already feels his brain is beyond all government. The allusion, however, by Coleridge, to the Fool's conclusion of this act by a " grotesque prattling," which he says " seems to indicate the dislocation of feeling that has begun, and is to be continued," is a striking instance of a man of genius wilfully choosing to confer something expressly of his own upon the passage he is criticising. The " grotesque prattling" is, in fact, a mere piece of farcical and indecent ribaldry to make the groundlings laugh, and was evidently inserted in accordance with the gross taste of the day, to which Shakspere certainly pandered far less than most of his contemporaries.* This is one of the worst instances. But the real conclusion of the act is in Lear's words last quoted.

* The *Fools* in Shakspere were considered of old as mere farcical claptraps, to " catch the ears of the groundlings;" and no doubt they really were intended as little else by Shakspere, and all the other dramatists of his time. The audiences of those days seem to have required some " fillip" of the kind during their long sittings of five or six hours at the playhouse. But critics of the present day have run into an opposite extreme, and are now endeavouring to lift some of the Fools of Shakspere into a prominence of a kind never intended by the poet—we mean, that of beauty, tenderness, and melancholy sentiment. The last instance of this has surpassed all preceding attempts, and may claim a few words of notice here, seeing that the Fool's utter want of true sympathy (which supposes sensitive perception) with the condition of Lear, is one of the chief incentives to his malady.

" In looking down the list of dramatis personæ to this play," says Mrs. Cowden Clarke, " we cannot but be struck with the *world of thought*, the *epitome of tenderness*, pity, attachment, gentleness, fancy," &c., &c., (we omit the rest of the long catalogue,) " all comprised in the image suggested to us by those four unpretending letters, F-o-o-l. No more formal announcement is deemed requisite to herald one of the *most lovely creations* that ever emanated from poet's brain," &c., &c. We are sorry to differ with so excellent and ingenious a lady as Mrs. Cowden Clarke, who, we think, has been led astray by her own amiable nature into an exaggerated view of this character, which the present essay will go far to display in a different colour.

The extent of ground gone over in the first Act of "King Lear" is quite extraordinary. Few other dramatists have lived who could have packed in fine order and clearness such a mass of diversified materials and movements in less than two or three acts. Nor could it have been done except by supposing a much longer interval to have transpired between Scene 1 and Scene 3 than is almost ever ventured by dramatists; nor, indeed, is it advisable for any one to attempt it. Here it has wonderfully succeeded. We are to remember that all the principal persons of the drama have been introduced; that we are already able to see and know each of their characters as clearly as if volumes had been written about them; and also that the characters and story of Gloster and his two sons are as definite, complete, and full of action and passion as those of Lear and his three daughters, of which they are at once the counterpart and underplot, distinct in themselves, yet almost inextricable from the main design.

In the interval of Act I. and Act II., we find the old king, having withdrawn himself from the immediate influence of the causes which were rapidly destroying his reason, has temporarily recovered himself, so that, when he enters at the opening of Scene 4, he seems to have a fair chance of regaining his self-government, (the little he ever possessed,) when the sight of his man in the stocks, and the refusal of Regan and Cornwall to speak with him, at present, excites his rage anew to a pitch with which he seems ready to burst.

> *Lear.* Vengeance! plague! death! confusion!
> Fiery?—What quality? Why, Gloster, Gloster,
> I'd speak with the Duke of Cornwall and his wife!
> *Gloster.* Well, my good lord, I have informed them so.
> *Lear.* Inform'd them! Dost thou understand me, man?

The indignity, as the ingratitude, is felt by Lear's passion to be so great, that he cannot understand it; he cannot believe that it can be really intended. His inordinate estimate of the kingly office, which he totally forgets he has surrendered, and his exactions as a father,—one, too, who was declared to be so dear that he had in return conferred half his kingdom, so far as land went, upon this daughter,—these things throw his feelings and thoughts into a dreadful conflict with the apparent external fact, and with themselves. He knows he is in the right, yet everything seems going wrong. He is confounded—he becomes frantic—he checks himself with an effort to be reasonable and make allowances—yet again he is driven wild.

> *Lear.* The *King* would speak with Cornwall; the *dear* father
> Would with his daughter speak,—*commands her service:*
> Are they inform'd of this? My breath and blood!
> Fiery? the fiery duke?—Tell the hot duke, that—
> No, but not yet; may be, he is not well, &c.

Chancing to turn to where Kent is seated in the stocks, he breaks off instantly, with "Death on *my state!*" His sense of regal dignity and power has received a shock, a blow that strikes at its very life,

His brain is in a whirl, and the roll and the hum of a ceaselessly beaten drum perfectly convey to us the maddening condition of his thoughts and emotions.

> *Lear.* Bid them come forth and hear me,
> Or at their chamber door I'll beat the drum
> Till it cry "Sleep to death!"

What a wild, fierce sense he has of the noise; he feels all the vibration in his brain—he informs the warlike instrument with his own frantic spirit, till it is ready to burst into a cry, which shall work a spell of deadly lethargy.

Nothing can be more strongly marked than the difficulty of the old King to understand or believe the extent of the wrong that is done him, and that it can really be intended. He gave away his kingdom with a distinct stipulation; he claims the performance as a right, independent of all he expected from love and duty; but instead of this love, this duty, and this right, he only meets with reproaches, defiance, and denial of all obligations. His utmost indignation and heaviest denunciations, instead of producing any shame or remorse in his unnatural daughters, only seem to stir them up to an attempt to show that *he* has done, and is constantly doing wrong, while they are everything that is noble, and reasonable, and right. "An inability," says Hazlitt, "to get rid of the distinction between right and wrong, is very likely to drive a man mad." This distinction constantly haunts Lear. The two ideas boil up alternately in his brain, till eventually they become fused; the distinction, though never lost to his passionate memory, is inextricably confounded, and disorder takes possession of his mind.

We must revert to our opinion first expressed, that Lear's madness, though displaying lurid gleams from the opening scene of the tragedy, becomes confirmed in the closing parts of the last scene of Act II. His curses on his daughters are terrific, and of a kind which no sane father, however injured, would utter. The elemental grandeur of his confusion of his own age with that of the heavens, has been finely noticed by Charles Lamb. His passions are so violent, that he expresses his sense of their explosive force by saying—"O sides, you are too tough—will you yet hold?" His imagination carries him from his inordinate sense of regality to wild extremes of savage life, where he shall become "a comrade with the wolf and owl," rather than return to Goneril with fifty men dismissed. He is very conscious of the condition into which he is falling. "I pr'ythee, daughter, do not make me mad!" From the extremes of violence he droops awhile into the weakness of dotage, where he says to Goneril, after all his dreadful imprecations, that he will go with her, because her fifty doubles Regan's five-and-twenty, and *therefore* she has twice her love! After this comes his terrible curses on them both, mixed with miserable tears, till he rushes out, exclaiming, "O Fool! I shall go mad!" And so it proves. The next time he appears (in Act III., Scene 2) it is on an open heath, in the midst of a storm, which only typifies that which rages within himself.

> *Lear.* Blow, winds, and crack your cheeks! rage! blow!

The Fool interposes with sundry motley remarks, among which, however, he tells the forlorn old King to "ask his daughters' blessing."

> *Lear.* Rumble thy bellyfull! spit fire! spout rain!
> Nor rain, wind, thunder, fire are my daughters!

There can be no doubt, as Ulrici remarks, that the mad state of Lear is, to a considerable degree, kept up by the poignant rodomontade of the Fool, though meant, perhaps, in kindness to lead the poor old King's mind away from too serious thoughts on the subject.

The storm of the elements evidently helps Lear to endure his own state; he feels a sympathy with it; it does him good; he compares it with his own inward tempest, and grows greater in himself by the comparison. He cares nothing for the storm without, until he suddenly bethinks him of his daughters.

> *Lear.* Pour on: I will endure.
> In such a night as this—O, Regan! Goneril!
> Your old kind father, whose frank heart gave all,—
> Oh, that way madness lies; let me shun *that*,—
> No more of *that*.

This consciousness of madness is quite a common thing with those who suffer under it. The effects of the storm, however, have considerably sobered the senses of the old King, and he is beginning to recover, as shown by the deeply pathetic and painfully wise prayer he offers up. It might be that this was destined to prove no more than a lucid interval, or that he might really have regained his reason from this point; but a new influence suddenly comes in to keep up his mental disorder.

"It is evident," says Dr. Hermann Ulrici, "that the overthrow of the King's senses is, partly, at least, occasioned by the crack-brained fancies with which the Fool keeps constantly mocking the folly of the King, although, no doubt, the assumed madness of Edgar powerfully co-operates in bringing it about. Thus, then, in this, as in every other detail, Shakspere combines the profoundest thought with the most artistic skill in furnishing adequate cause and motive for all that is said or done in his dramas." With all this we perfectly coincide, except that Lear's madness was not *occasioned* by the above; it was only re-illumined, kept up, and aggravated by them. Contemplating the almost-naked Edgar, the King thus speculates—

> Is man no more than this? Consider him well. Thou owest the worm no silk, the beast no hide, the sheep no wool, the cat no perfume: ha! there's three of us are sophisticated! Thou art the thing itself:—unaccommodated man is no more but such a poor, bare, forked animal as thou art. Off, off, you lendings!—Come, unbutton here! [*Tearing off his clothes.*]

Compare this with Lear's prayer previously, in the same scene, and the effect of his meeting with Edgar, or Mad Tom, will be at once apparent. The distinctions, however, between real madness and assumed madness are finely kept: though the two are thus strikingly brought in contact, they are never fused, (except by bad acting on the stage, where Edgar almost always makes the most he can of the madness, as if it were real;) nor can we mistake the extravagant incongruities and studied conceits of Edgar for the deep and pregnant aberrations of the *reason* which almost always accompany madness resulting from the wreck of strong passions and designs.

"In the aberrations of Lear's reason," says Charles Lamb, "we discover a mighty irregular power of reasoning, immethodised from the ordinary purposes of life, but exerting its powers, as the wind blows where it listeth, at will on the corruptions and abuses of mankind." This remark is strikingly true of Lear, but it is also a common characteristic of such a state. It should likewise be noticed as a fine distinction, that the more wild and preposterous the words and actions of Mad Tom, the more deep and speculative do the wandering faculties of the King become. Nor must we overlook the fact that some of the remarks of Gloster, on his own misfortunes, tend to aggravate what he would fain sooth in Lear.

*Edgar.* The Prince of Darkness is a gentleman:
Modo he's call'd, and Mahu.
*Gloster.* Our flesh and blood, my lord, is grown so vile,
That it doth hate what gets it.
*Edgar.* Poor Tom's a-cold.
*Gloster.* (*To Lear.*) Go in with me; my duty cannot suffer
To obey in all your *daughters' harsh commands,*
Though their injunction be to bar my doors
And let this tyrannous night take hold of you;
Yet have I ventured, &c.
*Lear.* First let me talk with this philosopher.
(*To Edgar.*) What is the *cause of thunder?*

The King takes the supposed madman aside for some serious private talk. The result confirms him in his hallucination—viz., that the apparent madman is a "learned Theban," a "noble philosopher," a "good Athenian." In the next scene, the same injurious, though well-meant, excitement is continued—

*Fool.* Prithee, nuncle, tell me, whether a madman be a gentleman or a yeoman?
*Lear.* A king! a king!
*Fool.* No; he's a yeoman, that has a gentleman to his son; for he's a mad yeoman that sees his son a gentleman before him.
*Lear.* To have a thousand with red burning spits
Come hissing in upon them:—
*Edgar.* The foul fiend bites my back.
*Fool.* He's mad that trusts in the tameness of a wolf, &c.
*Lear.* It shall be done, I will arraign them straight:—
(*To Edgar.*) Come, sit thou here, most learned justicer;—
(*To Fool.*) Thou, sapient sir, sit here. Now! you she-foxes!
*Edgar.* (*Aside.*) Look where he stands and glares!

Mr. Knight reads, "Look where *she* stands and glares," thus referring to *one* of the she-foxes, and in order, we suppose, to make the line correspond with the two which follow. We consider this an erroneous new reading, and that Edgar utters the line aside with reference to Lear. His doing so is quite in Shakspere's way, who thus continually gives us portraits of his characters under the variable aspects of their emotions, by a masterly, rapid, and scarcely perceptive touch as he passes. (Edgar's pity subsequently increases, and he says, aside, also, "Bless thy five wits!" and again, where he says his tears will mar his counterfeiting. The only wonder is, that he did not see cause for moderating his counterfeiting a little sooner.) But after making this observation aside, Edgar instantly resumes his character of Mad Tom, and addresses the two she-foxes alternately, as their "learned justicer."

> Wantonest thou eyes at trial, madam?
> Come o'er the bourne, Bessy, to me!

Be this, however, as it may, it can be no matter of surprise that the poor old King should "stand and glare" with all these helps and incentives. It is wonderfully kept up, (as a work of tragic art,) and most naturally, with the very best motives, and the most ignorant practice. Had Lear been placed under a quiet regimen, his mind might have regained its poise in a great measure. This is shown eventually. But at present, his fate is to have his disease wrought to the height. The Fool now sings; Mad Tom yells out about the foul fiend—Hopdance cries in his belly for two white herrings,—so that when Kent implores the King, half-deafened by the crazy fancies of Mad Tom on the one hand, and the Fool on the other, he only replies, "I'll see this trial first"—meaning his daughters'—and regards the ragged Mad Tom as the judge in his robes. Edgar and the Fool continue their jargon, humouring the King, as they mean it, but in the worst possible way for him—till the Fool, having evidently played some trick, by setting up a joint stool or other article, to represent Goneril placed at the bar, and then snatching it away, puts the same thing into the head of the King, who speaks of Regan in the same strain—as some wooden manufacture, which is suddenly snatched away—

> *Fool.* Cry you mercy, I took you for a joint stool.
> *Lear.* And here's another, whose *warp'd* looks proclaim
> What *store* her heart is made of:—Stop her there!
> Arms! arms! sword! fire! corruption in the place!
> False justicer, why hast thou let her 'scape!

Edgar now perceives that what they are about does the King no good, and feels the sight so pitiable, that he fears it will mar "his counterfeiting;" but it never occurs to him that he is contributing to the very malady he deplores. When Lear exclaims so pathetically—

> The little dogs and all,
> Tray, Blanch, and Sweetheart—see, they bark at me!

Edgar immediately replies that "Tom will throw his head at them;"

follows this up with a wild song about all sorts of dogs, and ends by pretending to suit the action to the word in the matter of the head—

> For, with throwing *thus* my head,

> Dogs leap the hatch, and all are fled!

Lear is wrought up to a corresponding pitch of mental aberration—gabbles in anguish and foolishness of anatomizing Regan—of entertaining the noisy Mad Tom for one of his hundred knights—objects to Mad Tom's rags, though conjecturing they are Persian attire—and then sinks down in sheer exhaustion, with a desire to escape from the turmoil, and a lethargic belief that the hard ground is a bed for the deepest repose—

> Make no noise, make no noise! draw the curtain! So, so, so! We'll go to supper in the morning. So, so, so!

In all this Edgar works the poor King's mind unbounded mischief. The secret of this lies in Edgar's deficiency in sensitive sympathy, not from want of feeling, but from the want of a sensitive imagination. He trifles with his old blind father in a different way, but to the same extent, even allowing him to attempt suicide, and to believe that he has actually fallen from Dover cliff! Edgar has acted mad extravagancies until they are beginning to take a strange sort of hold of him, and he cannot resist, even after this, telling his blind father that it was a fiend (though himself) with eyes like moons, whelk'd and ridgy horns, and "*a thousand noses,*" who had seduced him to the brink of the cliff!

Lear does not appear again till Scene 6 of the next Act (Act IV.), and what these previous scenes have done for him is but too wofully displayed. But before we come to this, let us see what Dr. Hermann Ulrici has to say on the wonderful condition of mind commonly called "madness."

"Madness is, as it were, the mind's revolt against itself—the loosening of the bonds between its subjectivity and objectivity, so that the two pass into each other, the merely subjective presentation (imagination) passing into objectivity, and the latter being transformed into merely subjective presentations."

Metaphysics is an abstruse and complicated science, and we are certainly not among those who, at the least sign of difficulty, or the use of peculiar terms, are ready to cry out "mystery" and "jargon," and so escape all further trouble. We think, however, the above might be more simply expressed—to the effect, that the impressions of external things become confused with the internal impressions or ideas, so that the imagination takes the place of outward substances and relations, while outward substances and relations become mere shadows and fancies. The definition is well worthy of study. We must, however, give the rest of the passage in Dr. Ulrici's own words, premising that it is his object to show that Shakspere has a moral and philosophical, and, above all, a religious design to work out in his tragedies. To the readers who are not previously acquainted with the passage we are about to give, (and we conjecture they are pretty numerous,) we think we may promise them something which they will be inclined to regard as an æsthetical, theological, and Fichtian curiosity.

"Every sin, consequently, must involve the germ of madness; for it is nothing less than the revolt of the mind from itself, and from its truth and objectivity in God. Nevertheless, as long as the sinner is able to maintain his *Ego*—which, in imagination, he has set up as the master both of himself and the world—in this untruth, so long does the delusion of sin appear outwardly as consistency, understanding, or truth; the madness remains as yet inclosed in the germ, and in its view of the world and of itself, the mind still preserves its adherence. When, however, through the might of circumstances, or the weakness of the body, which must supply the mind with food and vigour for its activity, the sinner's mental energy is broken, and he can no longer maintain his *Ego* in this fancied supremacy, while, at the same time, he is unable to cast off the strong fetters of his sin, and to throw himself upon the mercies of God, then does madness burst from the bud, and becomes total, both inwardly and outwardly. It appears no longer a revolt from God alone, but from itself and the world. The mind loses at once its organic centre of gravity, and is chaotically dissolved."

If Shakspere could but lift his sacred head from the tomb of ages, and read the foregoing passage "touching" his "King Lear," we are of opinion that the expression of countenance in the immortal bard would defy the powers of the most skilful portrait-painter extant. We cannot afford the space which would be requisite for the simplification of this philosophy, (sheer hallucination, we regard it, so far as Shakspere's *theological* and other *designs* are concerned,) and readers who may be indisposed to the labour, yet desirous to know more clearly what Dr. Ulrici is driving at, we must refer to some friend who is learned in German metaphysics, and a student of Fichte in especial. The conclusion is somewhat less recondite—

"This is why madness seizes the King and not Gloster. For Lear is 'every inch a king,'—had accustomed himself to be thought of, and set his heart on being the unlimited master of the world; although in boundless love he gives his kingdom away, it is still his sovereign pleasure to measure even affection by his own arbitrary will, and he would lord even over it. Even when he has overthrown his visionary empire by his own folly, he must still command; he fights against the very elements; he is determined to be, at least, the master of his own sufferings and his own destiny. But for this the necessary powers fail him, and consequently the general disorder of all the moral relations of life terminates in madness."

Amidst the heap of superficial criticism constantly afloat, under which no one has had more to suffer than Shakspere, nor perhaps half so much, it is at least satisfactory to see him approached with an earnest spirit and a profound study; and, however we may disagree with many of the conclusions of Dr. Ulrici, and particularly his laboured attempt to show that Shakspere had an ethico-theological design in all his great works, we ought to regard his labours with respect, and acknowledge the many fine new trains of speculation which he originates.

In Scene 4, Act IV., the equally graphic and pathetic picture of the poor mad Lear is rendered doubly affecting by the words which paint it, proceeding from the lips of Cordelia,—

> *Cordelia.* Alack, 'tis he;—why, he was met even now
> As mad as the vex'd sea: singing aloud;
> Crown'd with rank fumiter, and furrow weeds,
> With hurlocks, hemlocks, nettles, cuckoo-flowers,
> Darnel, and all the idle weeds that grow
> In our sustaining corn.

The physician being implored by Cordelia to do all he can for the restoration of her father's "bereaved sense," makes this reply,—

> *Physician.* There is means, madam!
> Our foster-nurse of nature is *repose,*
> *The which he lacks.*

He did indeed; for with the ceaseless gabble of the Fool on one side of him, and the outrageous over-acted vagaries of Mad Tom on the other, besides the storm of the elements, anything like a pause of quiet seemed out of the question, either by day or by night.

We are now fully prepared for what is to follow in Scene 6:—

> *Enter* LEAR, *fantastically dressed up with flowers.*
> *Lear.* No, they cannot touch me for coining;
> I am *the King himself.*
> *Edgar.* O, thou side-piercing sight!
> *Lear.* *Nature's above art, in that respect.*

Observe how subtly and characteristically abstract truth is at once inverted and confounded with practical truth in Lear's mind;—a king is a thing of Nature, and therefore above Art, just as the regal office is above an officer of the mint.

> *Lear.* Ha! Goneril with a white beard!"

It looks as if Lear suddenly said this on seeing Gloster; but we rather believe him to be thinking vaguely of himself and his lost identity; Goneril has taken possession of Lear—even of his white beard. He instantly proceeds to speak of himself as if in explanation of his injury:—

> *Lear.* They flattered me like a dog, and told me I had the white hairs in my beard ere the black ones were there. To say ay, and no, to everything I said: Ay, and no, too, was no good divinity. When the rain came to wet me once, and the wind to make me chatter; when the thunder would not peace at my bidding; there I found them, there I smelt them out. Go to, they are not men o' their words: they told me I was everything; 'tis a lie; I am not ague-proof.

*Gloster.* The trick of that voice I do well remember:
Is't not the king?
*Lear.* Ay, every inch a king!
When I do stare, see how the subject quakes!
I pardon that man's life: What was the cause?
Adultery?—
Thou shalt not die. Die for adultery! No!

In this wild yet pungent language, Lear shows that he recognises Gloster, or that something reminds his wandering senses of Gloster. Compare all this—and the profoundly searching and tranchant vein in which his imagination subsequently indulges—with the extravagant conceits and figures of Mad Tom, all superficial and of no meaning, and we shall discover what Charles Lamb calls Lear's "mighty irregular power of reasoning," and notice at the same time the marked distinction between real and assumed madness. There is nothing else so forcibly and fatally characteristic of aberration of mind as a continual tendency to reasoning and philosophizing on false premises, mal-a-propos, or with utter misapplication, and all this with a furor and intensity, or a morbid pertinacity, which is ready to stake life itself ten times over on the soundness of its logic. But that, amidst these wanderings, the deepest truths and principles are often enunciated, and generally with no more than a half-consciousness in the utterer, everybody who has conversed much with insane patients in asylums, or elsewhere, must certainly have observed.

The main principle of every theory of madness is comprised in the following brevity:—

*Lear.* No eyes in your head, nor no money in your purse? Your eyes are in a heavy case, your purse in a light: yet you see how this world goes.

*Gloster.* I *see* it *feelingly.*

*Lear.* What, *art mad?*

When a man sees everything, not as it is, but only through the medium of his personal feelings, he is undoubtedly in the first stage of madness, though the degrees will of course vary with the amount of passion (feeling) and the excitements of the imagination which accompany this morbid condition. To see external objects with the feelings rather than the organs of vision, is not exactly what Dr. Ulrici would call the exchange of places between the subjective and objective; but it is a similar process—viz., the mistaking internal for external senses. The conclusion of this speech, and the whole of the next, is a fine mixture of madness and philosophy:—

*Lear.* A man may see how this world goes, with no eyes. Look with thine ears: see how yon' justice rails upon yon' thief. Hark, in thine ear: change places; and handy-dandy, which is the justice, which is the thief? Thou hast seen a farmer's dog bark at a beggar?

*Gloster.* Ay, sir.

*Lear.* And the creature run from the cur? There thou might'st behold the great image of authority: a dog's obeyed in office.
Through tatter'd clothes small vices do appear;
Robes and furr'd gowns hide all. Plate sin with gold,

And the strong lance of justice hurtless breaks;
Arm it in rags, a pigmy's straw doth pierce it.
None does offend, none, I say, none. I'll able 'em!
Take that of me, my friend, who have the power
To seal the accuser's lips. Get thee glass eyes,
And like a scurvy politician, seem
To see the things thou dost not. Now, now, now,
Now!—pull off my boots! harder, harder; so.
*Edgar*. O, matter and impertinency mix'd!
Reason in madness.

It is curious enough, and natural enough, that the last remark should come from Edgar, who, in his assumed madness, had never thought of mixing any reasoning with it. He is now surprised to observe this—it had not struck him before.

*Lear*. If thou wilt weep my fortunes, take my eyes.
I know thee well enough; thy name is Gloster:
Thou must be patient; we came crying hither.
Thou know'st the first time that we smell the air,
We wawl and cry:—

A singularly expressive word is here coined for the occasion, as though the King imitated the *wawl* of a new-born child.

*Lear*. I will preach to thee—mark!

*Gloster*. Alack, alack the day!

The conjecture of Stevens is probably correct, that Lear here assumes the tone of a priest about to commence a sermon, but having taken off his hat, (the shape of which used to be called a "block,") his attention is caught by it, and his mind darts off to the fancy of shoeing a troop of horse with felt. We think he was led to this by the silence—a painful one—of those around him, which made him feel that even a horse, shod with felt, would be noiseless, and the better able to execute a sudden and secret vengeance.

*Lear*. When we are born we cry, that we are come
To this great stage of fools:—This a good block!
It were a *delicate stratagem* to shoe
A troop of horse with felt: I'll put it in proof;
And when I have stolen upon these sons-in-law,
Then kill, kill, kill, kill, kill!

The imagination of Lear, at the beginning and close of this scene, is evidently impressed with disconnected images of a battle-field, not only as a thing he longs for, but as if inspired by some wild presentiment of that which is about to happen in reality. He is so possessed with this, that when "a gentleman, with attendants," from Cordelia, come kindly to take him into their charge, he considers his battle has been fought, and the day lost:—

*Lear*. No rescue? What, a prisoner? I am even
The *natural fool* of fortune. Use me well;
You shall have reason. Let me have surgeons;
I am *cut to the brains*.

He presently makes an effort to recover himself and rise to his former dignity:—

> *Lear.* I will be jovial: come, come; I am a king,
> My masters, know you that?
> *Gentleman.* You are a royal one, and we obey you.
> *Lear.* Then there's life in 't.

The old overwrought feeling about the regal office. He had feared it was dead; but now, being obeyed, there's life in't.

The waking up of the old King, in Scene 7, Act IV., to a partial recovery of his senses, is not more profoundly affecting than profoundly true to nature under the distressing condition of fast failing faculties. It has all the uncertainty of a dream, with all the tenderness of a sweet nature which has been sorely abused, and now timidly seeks with trembling nerves to regain its position in some loving hearts.

> *Cordelia.* How does my *royal lord?* How fares *your majesty?*

How beautifully and tenderly Cordelia humours his weakness, though now, alas! it is too late. The same feeling operating in the first scene would have superseded the whole tragedy. By what fine and mingled threads do the works of nature and the highest genius hang in the air!

> *Lear.* You do me wrong, to take me out of the grave;—
> Thou art a soul in bliss; but I am bound
> Upon a wheel of fire, that mine own tears
> Do scald like molten lead.
> *Cordelia.* Sir, do you know me?
> *Lear.* You are a spirit, I know:—When did you die?
> *Cordelia.* Still, still, far wide!
> *Physician.* He's scarce awake; let him alone awhile,
> *Lear.* Where have I been?—Where am I?—Fair daylight?
> I am mightily abused—I should e'en die with pity,
> To see another thus. I know not what to say—
> I will not swear these are my hands; let's see:
> I feel this pin prick. Would I were assured
> Of my condition.
> *Cordelia.* O, look upon me, sir,
> And hold your hand in benediction o'er me:
> No, sir, *you must not kneel.*

We know of nothing more deeply pathetic than this in the whole range of tragedy. He who went mad through his inordinate sense of his dignity and self-will, now drooping on his knees to her whom he had "divorced from his love, and dowered with his hate."

> *Lear.* Pray, do not mock me:
> I am a very foolish, fond old man—
> Four score and upwards; not an hour more nor less.
> And to deal plainly,
> I fear I am not in my perfect mind.

Nor is he. His consciousness of this does not by any means alter the case. He has just said he was "fourscore and *upwards*," and fancied that this expressed his exact age to an hour.

*Lear.* Methinks I should know you—and know this man;
Yet I am doubtful; for I am mainly ignorant
What place this is; and all the skill I have
Remembers not these garments; nor I know not
Where I did lodge last night. Do not laugh at me;
For as I am a man, I think *this lady*
To be *my child*, Cordelia.

How timidly and tenderly he ventures on this belief in his own senses and memory.

*Cordelia.* And so I am—I am!
*Lear.* Be your tears wet? Yes, 'faith. I pray, weep not;
*If you have poison for me, I will drink it.*
I know you do not love me; for your sisters
Have, *as I do remember*, done me wrong:
You have some cause, they have not.

How touching his recognition of the injury he had done Cordelia. No more rage, and resentment, and violence; scarcely the memory of his wrongs. It is nearly all over with the old king.

*Cordelia.* No cause, no cause.
*Lear.* Am I in France?
*Kent.* In your own kingdom, sir.
*Lear.* Do not abuse me.

Cordelia says, "Wilt please your highness walk?" He scarcely understands her.

*Lear.* You must bear with me:
Pray you, now, forget and forgive: I am old and foolish.

In the foregoing Scene, and in the closing Scene of Act V., we see that the old King is worn out; his fury is all over; images presented to his mind, which at an early stage of his madness would have excited his passions to the utmost, now fall dead on his ear; his madness is on the verge of fatuity.

But before the lamp of passionate lunacy quite goes out, his energies flame up once more in the socket, and with his own hand and arm of "fourscore years and upwards," he kills the wretch who was murdering Cordelia. "Howl! howl! howl!" cries the mad old father, with the dead body of his daughter in his arms, borne onwards by supernatural strength. His mind is once more excited—insanity, in all its power, has again broken through the fast-closing vapour of time—its lurid gleam of anguish falls on the wan, cold face of his dead Cordelia, and is extinguished in scalding tears falling upon lips which have "no breath at all!"

On this great subject of "Madness, as treated by Shakspere," there is much more to say with regard to other tragedies; but we must defer it to a future opportunity.

